# Public Open Spaces and Leisure-Time Walking in Brazilian Adults

**DOI:** 10.3390/ijerph14060553

**Published:** 2017-05-23

**Authors:** Alex Antonio Florindo, Ligia Vizeu Barrozo, William Cabral-Miranda, Eduardo Quieroti Rodrigues, Gavin Turrell, Moisés Goldbaum, Chester Luiz Galvão Cesar, Billie Giles-Corti

**Affiliations:** 1 School of Arts, Sciences and Humanities, University of Sao Paulo, Sao Paulo 03828-000, Brazil; 2 Graduate Program in Nutrition in Public Health, Department of Nutrition, School of Public Health, University of Sao Paulo, Sao Paulo 01246-904, Brazil; eduquieroti@gmail.com; 3 Department of Geography, School of Philosophy, Literature and Human Sciences, University of Sao Paulo, Sao Paulo 05508-080, Brazil; lija@usp.br (L.V.B.); williamcabral@usp.br (W.C.-M.); 4 Institute for Health and Ageing, Australian Catholic University, Melbourne 3065, Australia; gavin.turrell@acu.edu.au; 5 Department of Preventive Medicine, School of Medicine, University of Sao Paulo, Sao Paulo 01246-903, Brazil; mgoldbau@usp.br; 6 Department of Epidemiology, School of Public Health, University of Sao Paulo, Sao Paulo 01246-904, Brazil; clcesar@usp.br; 7 Centre for Urban Research, Royal Melbourne Institute of Technology University, Melbourne 3000, Australia; billie.giles-corti@rmit.edu.au; 8 Adjunct, School of Population and Global Health, University of Melbourne, Melbourne 3010, Australia

**Keywords:** public open spaces, built environment, leisure-time walking, adults, Brazil

## Abstract

Access to public open space is important to increase leisure-time walking (LTW) in high-income countries, but there is little evidence in middle-income countries. We conducted a cross-sectional analysis to examine the relationship between LTW and the presence of different public open spaces (parks, bike paths, and squares) and the mix of these recreational destinations near the homes of adults participating in the Sao Paulo Health Survey (*n* = 3145). LTW was evaluated by a questionnaire. We delineated buffers (500, 1000, and 1500 m) from the geographic coordinates of the adults’ residential addresses using a geographic information system. We used multilevel logistic regression taking account of clustering by census tracts and households, and with adjustment for social, demographics, and health characteristics. The main results showed that the presence of at least two recreational destinations within a 500-m buffer of participants’ homes were associated with an increased odds of LTW compared with no destinations present (OR = 1.65; 95% CI 1.09–2.55). No associations were found for destinations further away. These results support actions outlined in the new urban plan for Sao Paulo city and could be used to highlight the importance access to a mix of public open spaces to promote physical activity in megacities of middle-income countries.

## 1. Introduction

Physical inactivity is a major global public health problem, now considered a pandemic that is associated with early mortality and with high public health care costs, and is an economic burden [1,2,3]. A publication with data from 142 countries showed that the cost to health care systems due to physical inactivity was about $53.8 billion in 2013, with 58% spent by the public sector [2]. Conversely, participation in physical activity—particularly physical activity during leisure time—is associated with improvements in well-being and quality of life in adults [4]. Yet, in middle-income countries, such as Brazil and Colombia, the prevalence of leisure-time physical activity among adults is lower [5] than high-income countries, such as the United States [6], and this situation has not changed in recent years [7,8].

Physical activity is a multifactorial behaviour. A systematic review showed that more than 70 factors have been associated with physical activity participation in people living in low and middle-income countries, including neighborhood environmental correlates [9]. Access to public open spaces, such as parks, green spaces, public plazas, and walking trails were found to be important for physical activity participation [10,11]. However, most studies conducted to date are in high-income countries [10,12], where the facilities, types of use, and built environment for physical activity may differ to low- and middle-income countries. For example, one study analyzing the built environment attributes in 15 cities in Australia, Belgium, Brazil, Colombia, the Czech Republic, Denmark, China, Mexico, New Zealand, Spain, and the United Kingdom showed large differences in walkability index sub-components, public transportation facilities, and recreation environments using 1000-m buffers [13].

Furthermore, results from studies in middle-income countries are inconsistent with significant within- and between-countries differences. Studies conducted in different cities in Brazil, for example, have not found associations between the presence or density of parks, bike paths, or public squares and leisure-time walking in adults [14,15]. Conversely, studies conducted in Colombia and Hong Kong have found positive associations between the density of parks and leisure time physical activity in adults or elderly people [16,17], while bike paths have been shown to be important in promoting leisure-time physical activity in Colombia [18].

In addition to studying whether different types of public open spaces are associated with physical activity, there is a need for evidence about whether the number and proximity of facilities are associated with physical activity. This type of evidence can be used to inform guidance about the design of active cities. Hence, researchers examine neighbourhoods around study participants’ homes by creating spatial-unit buffers [19]. These buffers range in size from between 400 m and 1600 m, and there is no consensus about the “ideal size” for physical activity promotion [13,19,20,21,22]. For example, a study conducted in Perth, Western Australia, showed that access to two or more different facilities for recreation in buffers of 1500 m increased the odds ratio for leisure-time walking of adults, but not in 400-m buffers [21]. Our evidence review found no studies that had examined the influence of the mix of different types of public open spaces, such as bike paths, parks, and squares within different buffer sizes on leisure-time walking for people living in low and middle-income countries, suggesting an important gap in the literature.

Sao Paulo is a megacity in Brazil that, in the last century, had an organic approach to city planning resulting in significant urban sprawl. However, in 2014, Sao Paulo approved a new urban plan that includes actions to improve public open spaces. In this case, it is important to verify if different types and the mix of the destinations in different buffer sizes are associated with health outcomes, like physical activity, because the answers to these questions can support the actions of the urban plan in Sao Paulo.

Therefore, the aims of this study were to examine the relationship between leisure-time walking with different types and mix of open spaces such as parks, squares, and bike paths in small and large buffers for people living in Sao Paulo, and whether these relationships differed by different size buffers.

## 2. Materials and Methods

### 2.1. Study Context, Design, and Sample

Sao Paulo is located in Southeastern Brazil, with a population in 2016 of 12,038,175 inhabitants living in 1521.10 km^2^ (population density = 7398 inhabitants per km^2^) [23]. Sao Paulo is the largest city and main economic centre in Brazil.

This study utilized the Sao Paulo Health Survey and was approved at the Ethical Committee of the School of Arts, Sciences and Humanities at the University of Sao Paulo (process number 55846116.6.0000.5390).

The Sao Paulo Health Survey is a cross-sectional study that has been conducted on three separate occasions (2003, 2008, and 2014–2015) and examines the health of adolescents and adults from Sao Paulo. The survey investigates different health behavior outcomes such as food consumption, nutrition status, physical activity, smoking, alcohol consumption, mental health, self-reported diseases, and use of health services. The survey data were collected by face-to-face interviews with adolescents (12–19 years old), adults (20–59 years old), and older adults (60 years or older).

This present study used the cross-sectional data of the Sao Paulo Health Survey conducted between August of 2014 and December of 2015. The sample was stratified in five health administrative areas of Sao Paulo (north, mid-west, southeast, south, and east) and a two-stage cluster sampling design was used. In the first stage, for each health administrative area 30 census tracts were selected (the smallest geographical unit for census purposes in Brazil), which totaled 150 for the entire city. In the second stage, 5940 households were randomly selected (an average of 39.6 per census tract). In final stage, the face-to-face interviews were conducted with 4043 study participants (12 years or older). This study used the adult sample only (i.e., *n* = 3406 people aged 18 years or more).

### 2.2. Outcome Variable

Leisure-time walking was the outcome in this study since 2003 the Sao Paulo Health Survey has collected physical activity behaviors using the International Physical Activity Questionnaire long version by face-to-face interview. This questionnaire has been validated for the Brazilian adult population and is frequently used in epidemiological studies in Latin American countries [24]. We create two binary outcomes: (1) participation in leisure-time walking (or not); and (2) 150 min or more per week of leisure-time walking (or not).

### 2.3. Covariates

Sex (men, women), age (in years), education (incomplete elementary school, elementary to incomplete high school, complete high school, undergraduate incomplete to complete), marital status (married or with partner, single, divorced or widower), body mass index (BMI) (% BMI ≥ 30 kg/m^2^; % BMI < 30 kg/m^2^), smoking (yes, no), and length of living at the same residence (in years) and residential region in Sao Paulo (north, mid-west, southeast, south, and east) were used to adjust for confounding in this study.

### 2.4. Public Open Spaces

Residential addresses were collected at interview. “Public Open Spaces” were defined broadly to include bike paths, squares, and parks. Data on the presence of these open spaces were derived from the georeferenced street dataset of the city, publicly available by the City Hall of Sao Paulo [25].

The independent variables were the presence of public open spaces (parks, bike paths, and squares) captured using varying sized buffers. We delineated radial buffers of 500, 1000, and 1500 m from the geographic coordinates of the adults’ residential addresses using a geographic information system (GIS) (Arc Map version 10.3, Redlands, CA, USA). These buffers represented the mean distances that an adult could walk between five and fifteen minutes [21].

We considered any size of open spaces (parks and squares) and any meter (bike paths) within of buffers of 500, 1000, and 1500 m. We calculated: (1) the presence or absence of each type of public open spaces within each buffer (yes or no); and (2) the mix of the presence or not of these destinations within each buffer size (0, 1, or ≥2 in buffers of 500 m and ≤1, 2, and 3 in buffers of 1000 and 1500 m).

### 2.5. Statistical Analysis

Data were analyzed with multilevel logistic regression using the xtmelogit command in Stata version 12.1 (StataCorp, TX, USA). We used two outcome variables: (1) leisure-time walking: yes or no; and (2) at least 150 min per week of leisure-time walking. The modelling was undertaken in two stages: Stage 1: we analyzed the association between leisure-time walking with each type of open space (bike paths, squares, and parks), and with the mix of open spaces at 500, 1000, and 1500 m taking account of clustering by census tracts and households, and with adjustment for sex, age (years), and area of residence (north, mid-west, southeast, south, east); Stage 2: further adjustment for education (incomplete elementary school, elementary school to incomplete high school, complete high school, undergraduate incomplete to complete), length living in the same residence (months), obesity (body mass index ≥ 30 kg/m^2^: yes or no), smoking (yes or no), and marital status (single, married or with partner, widowed or divorced). The results are presented as odds ratios and 95% confidence intervals.

## 3. Results

The geocoding of residential addresses was undertaken for 2246 households and the final sample for this study was *n* = 3145 adults (92.3% of total of 3406 adults that were interviewed in Sao Paulo Health Survey, mean of 1.4 adults and range of 1 to 14 adults interviewed by household). We had problems with geo-coding 187 residential addresses and, hence, 261 adults were excluded from the study.

Table 1 shows descriptive characteristics of the study participants. The majority were women, were 40 years or older (mean = 47.1 years, SD = 18.8 years), had lived in the same house for five years or more (mean = 17.7 years, SD = 15.4 years), were living with a partner or married, and half of the sample had completed high school or more. One-fifth were classified as obese (with a mean body mass index = 26.3 kg/m^2^, SD = 4.9 kg/m^2^) and 16% were smokers. Only one-fifth of participants walked for leisure and less than 10% achieved at least 150 min per week (mean = 39.7 min per week, SD = 166.3 min per week). The highest proportion of walkers were male, non-smokers, and people who were not obese. We had differences in the proportion of leisure-time walking according to education and age (≥150 min per week) (Table 1).

**Figure 1:**
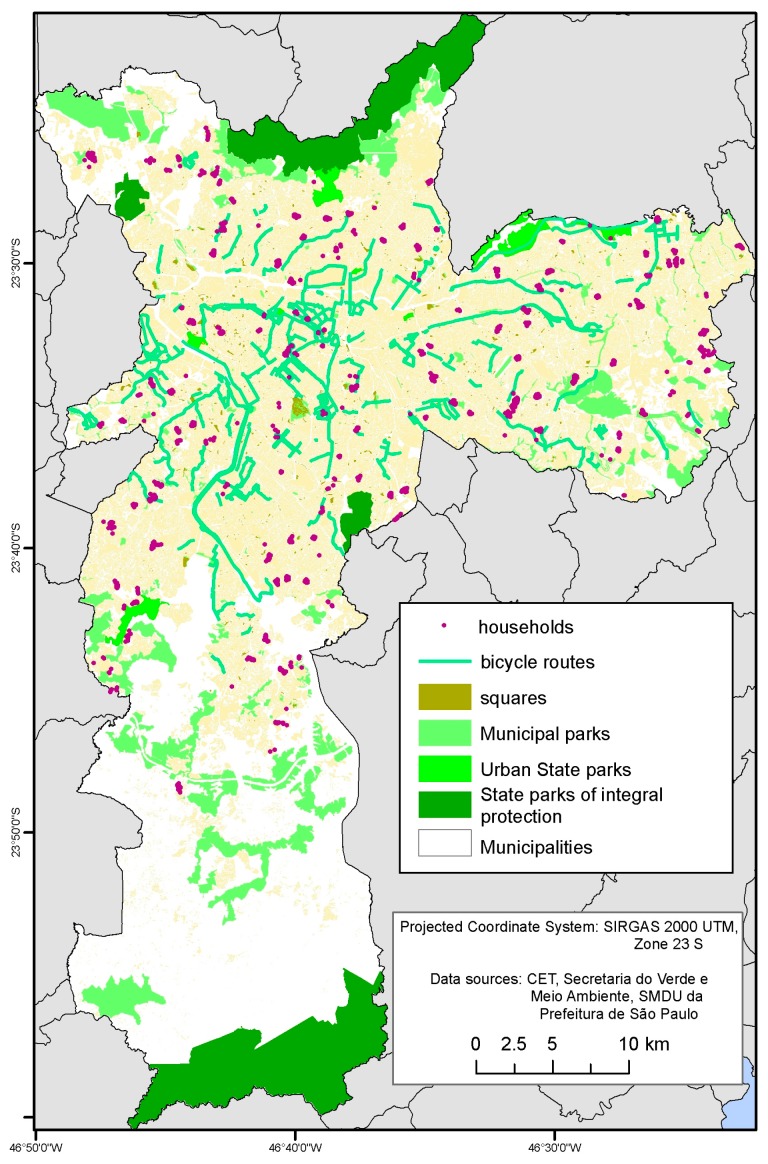
Public open spaces and households with interviews in Sao Paulo Health Survey, Sao Paulo, 2015.

The distribution of different open spaces varied around the city (Figure 1) and within the different sized buffers and across the sample (Table 2). Only 17.3% of participants had a park within a 500-m buffer of their homes, and 22% did not have any type of public open space within 500 m. However, most participants had squares or parks within a 1000-m buffer (>70%) and 1500-m buffer (>90%) and more than one-third in 1000-m buffers and half of participants had three or more destinations within a 1500-m buffer around their home.

The results of the multilevel model for each type of public open space showed that the odds of leisure-time walking was significantly higher for people living within 500-m buffers of a square or bike path (Table 3), but not a park. The results were independent of sex, age, and place where people living in Sao Paulo (model 1), and independent of education, marital status, obesity, smoking, and length of living in residence (model 2). The strongest effects were for the presence of bike paths: the odds of leisure-time walking was 55% higher for those living within 500-m of a bike path compared with those without a bike path (Table 3), and only the presence of bike paths within 500-m buffers increased the odds for regular walking (≥150 min per week). We found no significant association for any type of open space in 1000-m buffers or 1500-m buffers.

The results for the mix of destinations showed that participants who lived within 500-m buffers of two or more types of destinations had significantly higher odds of leisure-time walking (OR = 1.65; 95% CI 1.09–2.55) and for walking in leisure time for at least 150 min per week (OR = 1.66; 95% CI 1.03–2.69) compared with people who lived in buffers without any destinations (Table 4). The results were independent of sex, age, and region where people lived in Sao Paulo (model 1), and independent of education, marital status, smoking, and obesity (model 2). No associations were found for the 1000- and 1500-m buffers.

## 4. Discussion

This study showed that the presence of public squares, bike paths, and having at least two types of destinations within close proximity to people’s homes (i.e., a 500-m buffer) were associated with leisure-time walking in adults living in Sao Paulo. No associations were found for destinations further away (i.e., 1000-m and 1500-m buffers) highlighting the importance of close proximity.

There is no consensus among researchers regarding the relationship between the presence of open spaces near residences and leisure-time walking in adults living in middle-income countries. In adults of Curitiba, Brazil, the presence of squares or bike paths or parks in buffers of 500 m was not associated with walking in leisure time [14]. In Hong Kong however, Cerin et al. showed that the presence of parks in 400-m buffers were associated with walking for recreation in Chinese urban elders [17]. We showed that the presence of bike paths and squares in close proximity to residences (500 m) was associated with leisure-time walking. However, no significant association was found for parks further away.

Access to bike paths appeared to be important for encouraging physical activity in Sao Paulo. Currently, the city has more than 400 km of bike paths and many are shared between cyclists, pedestrians, and runners. Having proximate squares also appeared important for physical activity with the proportion of proximate squares in Sao Paulo being higher than parks. Notably, we found no association between park access and leisure-time walking. This is surprising because green areas, such as parks, often have more facilities than squares, including trails for walking, trees, and equipment for physical exercise. Hence, other factors other than just proximity may be important to encourage leisure-time walking. For example, in Perth, Australia, a study published in 2008 found no association between leisure-time walking and the presence of parks in buffers of 400 m [21]. However, another later study conducted in this same city showed that highly attractive parks near residences (within median 471 m) (i.e., those aesthetically pleasing, safe, and amenities present) were associated with leisure-time walking in adults [26]. It could be that our non-significant findings in Sao Paulo, may be due to the fact that we only measured proximity, and ignored the size and attractiveness of the parks, which others have found to be important [26].

The mix of different types of recreational destinations within 500-m buffers was associated with leisure-time walking in adults of Sao Paulo, but not in 1000-m buffers and 1500-m buffers. These findings are also in contrast to those in Perth, Australia, where the mix of recreational destinations within buffers of 1500 m was associated with walking during leisure time, but not in the smaller buffers of 400 m [21]. The results from Sao Paulo suggest that having some facilities in close proximity of residences may be more important than the number of facilities further away (1500-m buffers). However, only 32% of this sample from Sao Paulo city had at least two types of recreational physical activity destinations in close proximity to their homes. These results suggest that increasing the number of local recreational destinations, such as public squares and bike paths, within close proximity to residences in Sao Paulo may be important for promoting increased physical activity.

No type of public open space was associated with leisure-time walking in 1000-m and 1500-m buffers. A number of factors may explain these results. In these larger buffers we had very low variation in access to parks and squares (i.e., nearly 90% people had access within 1500-m buffers). Nevertheless, we also found no significant association between the presence of bike paths and leisure-time walking in 1000-m and 1500-m buffers, despite good variation in access present (52% and 57% of people had access to these facilities in 1000-m buffers and 1500-m buffers, respectively) and the association observed within 500-m buffers were both significant and strong.

However, it could be that the radial buffer applied in this study may have introduced measurement error [10,22]. Another study that used network buffers showed that the leisure-time walking was associated with the mix of recreational and utilitarian destinations in 1500-m buffers in Perth, Australia [27]. Nevertheless, at this stage of its development of geospatial data, it was not possible to create network buffers in Sao Paulo.

This study has other limitations. Firstly, the cross-sectional study design does not allow us to know the temporality of the relationship between open spaces with leisure-time walking and we may have observed problems associated with reverse causality, mainly because we do not control for neighborhood self-selection [28]. In the case of bike paths, many kilometers in Sao Paulo have been built since June 2014 and the physical activity data collected by survey was between August 2014 and December 2015. However, the problems with neighborhood self-selection have decreased because most of the people had lived in the same house for five years or more. The prevalence of the walking in leisure time, the outcome in this study, was similar with a survey that was conducted with adults in all capitals in Brazil including Sao Paulo [29], and the analysis in this study controlled for many demographic, social, and health factors that might confound the association between the features of the built environment and walking. Nevertheless longitudinal studies are still needed because causality cannot be implied from cross-sectional studies. Secondly, the analysis of this study was only based on the presence or absence, and in the mix of, the public open spaces within differently-sized buffers, with no consideration of other potentially important factors. People may choose the facilities not only by proximity, but also attractiveness, including aesthetics, safety, amenities, and their size [26].

Finally, this study did not use a weighted sample. In this case, some demographic characteristics of the Sao Paulo Health Survey sample were different from the Sao Paulo population. According to census data of 2010 [23], Sao Paulo comprises 52.6% of women and 16.6% of people 60 years or older, i.e., a lower prevalence than observed in the sample of this study. In addition, 20.5% of the Sao Paulo population aged 25 years or older have completed undergraduate education [23], but in the sample of this study, only 16% had the same level of education (data not shown). However, with adjustment for covariates, the results were independent of sex, age, and of other social and health characteristics as education, marital status, length living in the same residence, obesity, smoking, and place where people lived in Sao Paulo.

Despite some limitations, the results of this study showed that the presence of bike paths and squares and the mix of recreational destinations within 500-m buffers around the homes of residents appear to be important for physical activity promotion in a megacity such as Sao Paulo. This distance makes sense for some cities in Brazil. The quasi-experimental study conducted in Florianopolis, a city from the south of Brazil that showed that the introduction of walking trails contributed to an increase of leisure-time walking of people living within 500-m of this facility [30]. Additionally, in 2014, a new urban development plan for Sao Paulo was approved. Green areas and facilities, such as bike paths, were included as part of this plan and these will be increased and improved around the city in the coming years, providing an opportunity to monitor the impact on the physical activity patterns of residents over time.

## 5. Conclusions

Results from Sao Paulo indicate that the presence of squares and/or bike paths, and having two or more types of recreational destinations within 500 m of home, increases leisure-time walking participation in adults. These results are important because they support actions outlined in Sao Paulo’s new urban plan aimed at increasing access to public open spaces. They highlight the importance of monitoring the health and wellbeing impacts of this new urban policy. The results also highlight the potential for the research findings to foster discussions among researchers and policy-makers and how evidence might be used to highlight the importance of open spaces to promote physical activity in megacities of middle-income countries, such as Sao Paulo.

## Figures and Tables

**Table 1:** Descriptive statistics of general sample and for participants that reported leisure-time walking, Sao Paulo, 2014–2015.

Variables	Total Sample (*n* = 3145)	Any Leisure-Time Walking		Leisure-Time Walking ≥150 min per Week	
	%	%	*p*	%	*p*
*Sex*			0.008 *		0.015 *
Men	42.5	24.2		9.8	
Women	57.7	20.3		7.4	
*Age*			0.519		0.048 *
18–29	22.4	22.7		7.4	
30–39	17.6	21.0		8.3	
40–49	15.0	18.9		6.4	
50–59	14.2	21.7		8.5	
60 or older	30.9	23.5		10.1	
*Education*			<0.001 *		0.002 *
Incomplete elementary school	24.9	16.8		6.8	
Elementary to incomplete high school	25.1	20.6		7.4	
Complete high school	27.7	21.1		8.6	
Undergraduate incomplete to complete	22.3	30.6		11.1	
*Marital Status*			0.259		0.368
Married or with partner	54.1	20.5		8.6	
Single	26.7	25.6		9.0	
Divorced or widower	19.2	21.2		7.1	
*Length of residence*			0.859		0.083
≤1 year	11.0	21.4		5.6	
>1 year and <5 years	18.5	23.4		8.7	
>5 years	70.5	21.9		8.8	
*Smoking*			0.001 *		0.072
Yes	16.0	16.3		6.4	
No	84.0	23.0		8.8	
*Body Mass Index (BMI)*			<0.001 *		0.032
% BMI ≥ 30 kg/m^2^	22.1	16.3		6.4	
% BMI < 30 kg/m^2^	77.9	23.5		9.0	
*Leisure-time walking*					
% yes	21.9	-		-	
% ≥150 min per week	8.4	-		-	

* *p*-value < 0.05 according to chi-square test.

**Table 2:** Descriptive statistics of the destination to open spaces for geocoded sample according to buffer size, Sao Paulo, 2015.

Respondents with Destination within Buffers (*n* = 3145 Respondents)	%
Destination within 500-m buffers	
Bike paths	29.3
Squares	66.4
Parks	17.3
Destination within 1000 m buffers	
Bike paths	52.4
Squares	87.0
Parks	73.6
Destination within 1500-m buffers	
Bike paths	57.7
Squares	94.1
Parks	92.2
Mix of destinations in 500-m buffers	
0	22.1
1	46.3
≥2	31.6
Mix of destinations in 1000-m buffers	
≤1	22.5
2	39.7
3	37.8
Mix of destinations in 1000-m buffers	
≤1	9.4
2	37.0
3	53.5

**Table 3:** Odds ratios (OR) for the association between participating in walking for leisure and types of destinations within buffers of 500 m, 1000 m, and 1500 m.

Types of Destinations within Buffers	Leisure-Time Walking (Any Walking vs. No Walking)		Leisure-Time Walking (≥150 min/Week vs. <150 min/Week)	
	Model 1 OR (95% CI)	Model 2 OR (95% CI)	Model 1 OR (95% CI)	Model 2 OR (95% CI)
**500-m buffers**				
Squares				
No	1	1	1	1
Yes	1.44 (1.03–2.02) *	1.41 (1.00–1.97) *	1.36 (0.94–1.96)	1.29 (0.89–1.89)
Bike paths				
No	1	1	1	1
Yes	1.63 (1.16–2.29) *	1.55 (1.11–2.16) *	1.59 (1.12–2.27) *	1.53 (1.07–2.18) *
Parks				
No	1	1	1	1
Yes	1.02 (0.69–1.51)	0.97 (0.66–1.43)	1.00 (0.64–1.52)	0.99 (0.64–1.53)
**1000-m buffers**				
Squares				
No	1	1	1	1
Yes	0.98 (0.61–1.57)	1.00 (0.62–1.59)	1.01 (0.61–1.67)	0.97 (0.57–1.65)
Bike paths				
No	1	1	1	
Yes	1.26 (0.90–1.76)	1.22 (0.88–1.70)	1.15 (0.80–1.64)	1.13 (0.79–1.63)
Parks				
No	1	1	1	1
Yes	0.94 (0.66–1.32)	1.00 (0.71–1.41)	0.77 (0.53–1.10)	0.82 (0.57–1.19)
**1500-m buffers**				
Squares				
No	1	1	1	1
Yes	0.78 (0.42–1.47)	0.85 (0.45–1.61)	0.67 (0.35–1.31)	0.72 (0.36–1.43)
Bike paths				
No	1	1	1	1
Yes	1.26 (0.91–1.76)	1.26 (0.91–1.74)	1.31 (0.93–1.86)	1.28 (0.89–1.82)
Parks				
No	1	1	1	1
Yes	1.42 (0.78–2.60)	1.57 (0.86–2.87)	0.83 (0.45–1.51)	0.87 (0.47–1.59)

Model 1 adjusted by sex, age, and the residential region in Sao Paulo; Model 2 adjusted by sex, age, education, marital status, obesity, smoking, length living in residence, and residential region in Sao Paulo; * *p* < 0.05.

**Table 4:** Odds ratios (OR) for the association between participating in leisure-time walking and the mix of the types of destinations within buffers of 500 m, 1000 m, and 1500 m.

Mix of Types of Destinations	Leisure-Time Walking (Any Walking vs. No Walking)			Leisure-Time Walking (≥150 min/Week vs. <150 min/Week)		
	Prevalence (%)	Model 1 OR (95%CI)	Model 2 OR (95%CI)	Prevalence (%)	Model 1 OR (95%CI)	Model 2 OR (95%CI)
500-m buffers						
0	18.9	1	1	6.8	1	1
1	20.2	1.07 (0.72–1.59)	1.08 (0.73–1.72)	7.5	1.08 (0.69–1.69)	1.05 (0.66–1.67)
≥2	26.6	1.73 (1.13–2.66) *	1.65 (1.09–2.55) *	10.9	1.73 (1.08–2.76) *	1.66 (1.03–2.69) *
1000-m buffers						
≤1	18.5	1	1	8.2	1	1
2	23.2	1.26 (0.85–1.86)	1.24 (0.84–1.83)	8.6	1.00 (0.66–1.53)	1.01 (0.65–1.55)
3	22.6	1.18 (0.77–1.80)	1.21 (0.80–1.85)	8.3	0.90 (0.57–1.42)	0.92 (0.58–.1.46)
1500-m buffers						
≤1	15.9	1	1	7.8	1	1
2	20.5	1.36 (0.76–2.42)	1.31 (0.73–2.35)	7.6	0.87 (0.47–1.62)	0.95 (0.50–1.82)
3	24.0	1.47 (0.83–2.63)	1.52 (0.84–2.69)	9.0	1.00 (0.54–1.84)	1.06 (0.56–2.00)

Model 1 adjusted by sex, age, and the residential region in Sao Paulo; Model 2 adjusted by sex, age, education, marital status, obesity, smoking, length living in residence, and the residential region in Sao Paulo; * *p* < 0.05.
